# Combined targeting of mTOR and c-MET signaling pathways for effective management of epithelioid sarcoma

**DOI:** 10.1186/1476-4598-13-185

**Published:** 2014-08-07

**Authors:** Yoshinori Imura, Hirohiko Yasui, Hidetatsu Outani, Toru Wakamatsu, Kenichiro Hamada, Takaaki Nakai, Shutaro Yamada, Akira Myoui, Nobuhito Araki, Takafumi Ueda, Kazuyuki Itoh, Hideki Yoshikawa, Norifumi Naka

**Affiliations:** Department of Orthopaedic Surgery, Osaka University Graduate School of Medicine, 2-2 Yamadaoka, Suita, Osaka, 565-0871 Japan; Musculoskeletal Oncology Service, Osaka Medical Center for Cancer and Cardiovascular Diseases, 1-3-3 Nakamichi, Higashinari-ku, Osaka, 537-8511 Japan; Department of Orthopaedic Surgery, Osaka National Hospital, 2-1-14 Hoenzaka, Chuo-ku, Osaka, 540-0006 Japan; Department of Biology, Osaka Medical Center for Cancer and Cardiovascular Diseases, 1-3-3 Nakamichi, Higashinari-ku, Osaka, 537-8511 Japan

**Keywords:** Epithelioid sarcoma, mTOR, c-MET, AKT, ERK, RAD001, INC280

## Abstract

**Background:**

Epithelioid sarcoma (EpS) is a high-grade malignant soft-tissue sarcoma characterized by local recurrences and distant metastases. Effective treatments for EpS have not been established and thus novel therapeutic approaches against EpS are urgently required. mTOR inhibitors exert antitumor effects on several malignancies but AKT reactivation by mTOR inhibition attenuates the antitumor effects of mTOR inhibitors. This reactivation is receptor tyrosine kinase (RTK)-dependent due to a release of negative feedback inhibition. We found that c-MET was the most highly activated RTK in two human EpS cell lines, Asra-EPS and VAESBJ. Here we investigated the functional and therapeutic relevance of mTOR and/or c-MET signaling pathways in EpS both *in vitro* and *in vivo*.

**Methods:**

We first examined the effects of an mTOR inhibitor, RAD001 (everolimus), on cell proliferation, cell cycle, AKT/mTOR signaling, and xenograft tumor growth in EpS cell lines. Next, we determined whether RAD001-induced AKT reactivation was blocked by silencing of c-MET or treatment with a selective c-MET inhibitor, INC280. Finally, we evaluated the antitumor effects of RAD001 combined with INC280 on EpS cell lines compared with either single agent or control *in vitro* and *in vivo*.

**Results:**

Constitutive AKT phosphorylation was observed in Asra-EPS and VAESBJ cells. RAD001 suppressed EpS cell growth by inducing cell cycle arrest but enhanced AKT phosphorylation, which resulted in intrinsic resistance to mTOR inhibitors. In both EpS cell lines, RAD001-induced AKT phosphorylation was dependent on c-MET signaling. INC280 inhibited phosphorylation of c-MET and its downstream molecules, and decreased RAD001-induced phosphorylation of both AKT and ERK in EpS. Compared with a single agent or control, the combination of RAD001 and INC280 exerted superior antitumor effects on the growth of EpS cell lines *in vitro* and *in vivo*.

**Conclusions:**

Targeting of mTOR and c-MET signaling pathways significantly abrogates the growth of EpS in preclinical models and may be a promising therapeutic approach for patients with EpS.

**Electronic supplementary material:**

The online version of this article (doi:10.1186/1476-4598-13-185) contains supplementary material, which is available to authorized users.

## Background

Epithelioid sarcoma (EpS) was first described in 1970 by Enzinger as a distinct soft-tissue tumor with mixed epithelial and mesenchymal phenotype [1], but the origin and true nature of EpS remain controversial. In general, EpS is relatively rare and accounts for less than 1% of all soft-tissue sarcomas [2]. The overall 5-year survival rates are 32%–78% [2–5]. The clinical course of EpS is usually characterized by local recurrences and distant metastases to lymph nodes and lungs, but an effective chemotherapy has not yet been established [2–5]. Therefore, novel therapeutic approaches against EpS are critically needed.

The phosphatidylinositol 3-kinase (PI3K)/AKT/mTOR signaling pathway, which drives cell proliferation, motility, and survival, is frequently hyperactivated in a variety of malignancies [6, 7], and inhibition of this pathway has been considered an appropriate approach for cancer therapy. Integrase interactor 1 (INI-1) is the protein product of the tumor suppressor gene *hSNF5/INI1/SMARCB1/BAF47* located on 22q11.2. Loss of INI-1 serves as a diagnostic feature in malignant rhabdoid tumors (MRTs) and atypical teratoid/rhabdoid tumors (AT/RTs) [8, 9]. Darr and colleagues reported that INI-1-deficient tumor cells exhibited persistent activation of AKT signaling [10]. INI-1 expression is also lost in most EpS clinical samples [11, 12], suggesting that AKT signaling may also be activated in EpS cells. In the present study, we detected loss of INI-1 expression and constitutive AKT activation in two human EpS cell lines, Asra-EPS [13] and VAESBJ [14].

AKT activation has been proposed as a predictor of response to rapamycin, which is an allosteric mTOR inhibitor [15]; this concept raises the possibility that mTOR inhibitors may be effective on EpS. Administration of these drugs results in reduction of regulatory proteins involved in progression of cells from the G1 to S-phase of their growth cycle [16]. The U.S. Food and Drug Administration has approved mTOR inhibitors for treatment of neuroendocrine tumors, renal cell carcinoma, and subependymal giant cell astrocytoma associated with tuberous sclerosis. However, the antitumor effects of mTOR inhibitors on patients with bone or soft-tissue sarcomas are limited, and responses are frequently short lived [17, 18]. In addition, blocking mTOR activity inadvertently reactivates AKT signaling, which mitigates the antitumor effects of mTOR inhibitors, and this reactivation has been posited as a mechanism of intrinsic resistance to mTOR inhibitors [19–22].

The AKT/mTOR signaling pathway is normally regulated by upstream receptor tyrosine kinases (RTKs) [23–25]. The resistance to mTOR inhibitors has been reported to be caused by RTK-dependent AKT reactivation due to a release of negative feedback inhibition [19–22]. Overexpression of hepatocyte growth factor (HGF) and its receptor, known as c-MET, is observed in most EpS clinical samples [26]. We demonstrated that c-MET was highly activated via an autocrine HGF loop in both EpS cell lines. The HGF/c-MET signaling pathway is critical in cell proliferation, motility, and invasion of several human sarcomas [27–29], but little is known about its biological functions in EpS.

In the present study, we first examined the therapeutic efficacy of an mTOR inhibitor, RAD001 (everolimus; Novartis Pharma AG, Basel, Switzerland), on two human EpS cell lines, Asra-EPS and VAESBJ. Next, we investigated whether RAD001-induced AKT reactivation was dependent on c-MET signaling. Finally, to seek a novel therapeutic modality for EpS, we evaluated the antitumor effects of combining RAD001 with a c-MET inhibitor, INC280 (Novartis Pharma AG), on the growth of EpS cell lines *in vitro* and *in vivo*.

## Results

### The AKT/mTOR pathway is constitutively hyperactivated in EpS

**Figure 1:**
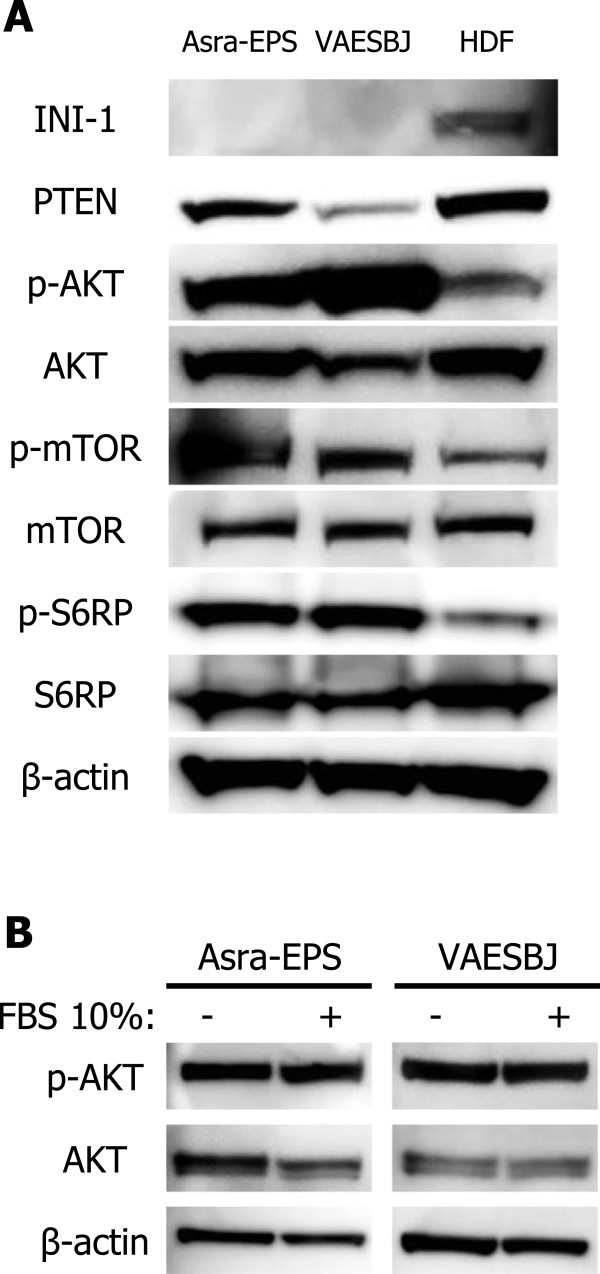
**The AKT/mTOR pathway is constitutively activated in Asra-EPS and VAESBJ cell lines. A)** Expression of proteins related to the AKT/mTOR pathway in Asra-EPS, VAESBJ, and HDF cells. **B)** Expression of p-AKT in the absence or presence of serum in Asra-EPS and VAESBJ cells. Cells were seeded in normal growth medium, grown over night, and incubated in the absence or presence of serum for 12 hours.

To investigate whether the AKT/mTOR pathway was activated in EpS, we examined the expression of its related molecules in Asra-EPS and VAESBJ cells. AKT, mTOR, and S6 ribosomal protein (S6RP) were more intensely phosphorylated in Asra-EPS and VAESBJ cells than in human dermal fibroblast (HDF) cells (KF4009; Kurabo, Osaka, Japan), while INI-1 expression was completely lost in both EpS cells (Figure 1A). These data indicated that the AKT/mTOR pathway was hyperactivated in EpS. Further, AKT phosphorylation was stronger in VAESBJ cells than in Asra-EPS cells. PTEN, which negatively regulated the AKT pathway [30], was less expressed in VAESBJ cells (Figure 1A), suggesting the possibility of marked AKT phosphorylation in these cells. We observed that AKT phosphorylation was evident even in the absence of serum in both EpS cell lines (Figure 1B), indicating that EpS cells had an aberrant and constitutive activation of AKT signaling.

### RAD001 suppresses EpS cell growth but enhances AKT activation

**Figure 2:**
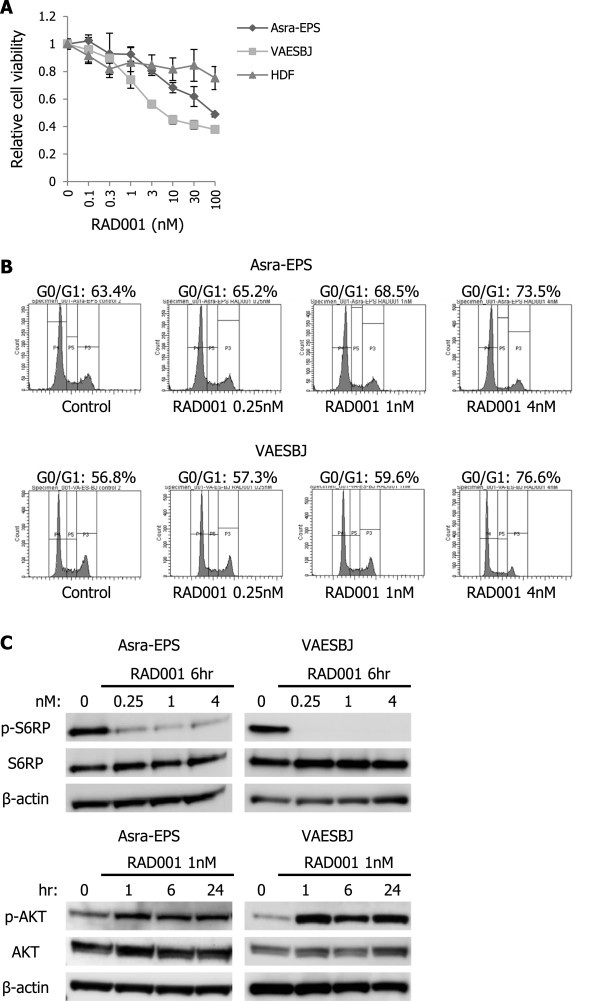
**RAD001 inhibits EpS cell growth but increases AKT activation** ***in vitro*** **. A)** Sensitivities of Asra-EPS, VAESBJ, and HDF cells to RAD001. Cells were exposed to various concentrations of RAD001 for 72 hours. Cell viability was determined by WST-1 assay. Points, mean; bars, SD. **B)** PI staining fluorescence-activated cell sorting analyses of the DNA contents of Asra-EPS and VAESBJ cells in response to RAD001. Cells were treated with 0.25–4 nM of RAD001 or vehicle for 24 hours. **C)** Effects of RAD001 on phosphorylation of S6RP and AKT in Asra-EPS and VAESBJ cells. Cells were treated with 0.25–4 nM of RAD001 or vehicle for 6 hours and with 1 nM RAD001 for 1–24 hours.

To assess the functional role of the AKT/mTOR pathway in EpS, we first tested the effects of an mTOR inhibitor, RAD001, on EpS cell proliferation *in vitro*. RAD001 treatment induced a dose-dependent decrease in the proliferation of EpS cells compared with no significant change in that of HDF cells (Figure 2A). To investigate whether RAD001 inhibited EpS cell proliferation by blocking mTOR signaling, we transfected two kinds of anti-mTOR-specific siRNAs into each EpS cell line and examined the effects of mTOR silencing. The expression of mTOR and p-mTOR was inhibited by anti-mTOR siRNAs in both EpS cells (Additional file 1: Figure S1A). In addition, the silencing of mTOR expression suppressed VAESBJ cell proliferation and decreased the anti-proliferative effect of RAD001 on VAESBJ cells (Additional file 1: Figure S1B, C). These data suggested that the AKT/mTOR signaling pathway affected EpS cell growth and that RAD001 inhibited EpS cell proliferation by blocking this pathway. RAD001 exposure increased G0/G1 phase population and decreased S phase population in a dose-dependent manner in both EpS cell lines (Figure 2B), implying that the antitumor mechanism of RAD001 primarily appeared to be exerted by G0/G1 cell cycle arrest. Western blot analyses showed that S6RP phosphorylation was inhibited after RAD001 treatment in EpS but that AKT phosphorylation was increased (Figure 2C). These results demonstrated that RAD001 blocked the mTOR pathway but enhanced AKT activation in human EpS.

**Figure 3:**
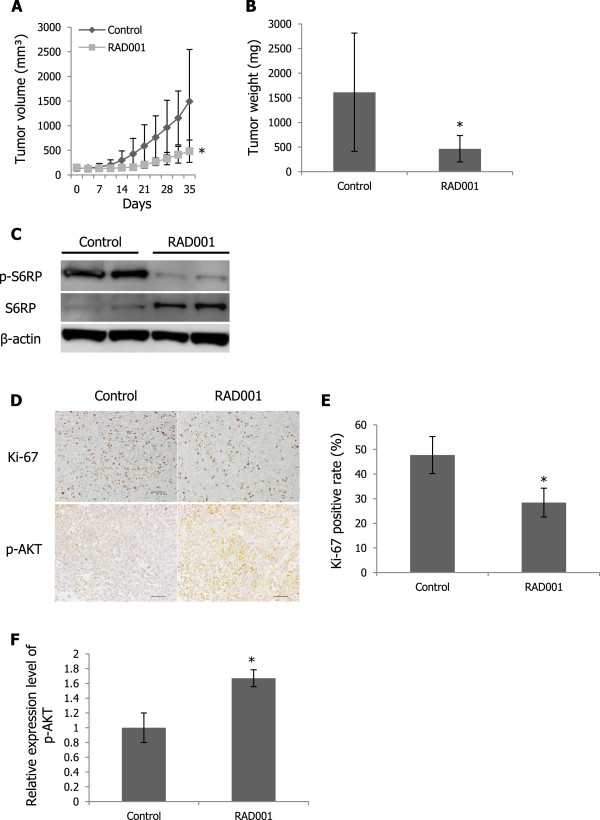
**RAD001 delays Asra-EpS xenograft tumor growth but enhances AKT activation** ***in vivo*** **. A)** Effects of RAD001 on Asra-EPS xenograft tumor growth. Nude mice bearing Asra-EPS xenograft tumors were treated with 5 mg/kg RAD001 (n = 5) or vehicle control (n = 5) thrice a week. Points, mean; bars, SD. *, p < 0.05, compared with control treatment. **B)** Asra-EPS xenograft tumor weights in control-treated and RAD001-treated mice. The average tumor weight recorded at termination of the study was 1613 ± 1200 mg in the control group and 466 ± 269 mg in the RAD001 group. Columns, mean; bars, SD. *, p < 0.05, compared with control treatment. **C)** Effects of RAD001 on S6RP phosphorylation in Asra-EPS xenograft tumors. Tumors were harvested 3 hours after the last administration and then cell lysates were prepared. **D)** Immunohistochemical staining of Ki-67 and p-AKT in control-treated and RAD001-treated Asra-EPS xenograft tumors. Scale bars: 100 μm. **E)** Ki-67-positivity rate of control-treated and RAD001-treated Asra-EPS xenograft tumors. Columns, mean; bars, SD. *, p < 0.05, compared with control treatment. **F)** Relative expression levels of p-AKT in control-treated and RAD001-treated Asra-EPS xenograft tumors. Relative expression levels were normalized against control-treated tumors. Columns, mean; bars, SD. *, p < 0.05, compared with control treatment.

Subsequently, we evaluated the antitumor effects of RAD001 on Asra-EPS xenograft tumor growth in nude mice. The growth rate of Asra-EPS xenograft tumors was delayed in RAD001-treated mice compared with that in control-treated mice, although RAD001 treatment did not induce tumor shrinkage (Figure 3A, B). Western blot analyses exhibited a decrease in expression of phosphorylated S6RP in RAD001-treated xenograft tumors (Figure 3C), indicating that the mTOR signaling was indeed blocked *in vivo*. Next, formalin-fixed and paraffin-embedded tumor sections from mice of both study arms were immunohistochemically evaluated. A decrease in the rate of Ki-67-positive tumor cells and an increase in expression of phosphorylated AKT were observed in RAD001-treated tumors (Figure 3D–F). These data showing tumor growth delay without shrinkage suggested that RAD001-induced AKT reactivation may decrease the activity of RAD001 and that agents blocking this reactivation could enhance the antitumor effects of RAD001 on the growth of EpS.

### c-MET is highly activated through an autocrine mechanism in EpS cells, and c-MET activation contributes to EpS cell growth

**Figure 4:**
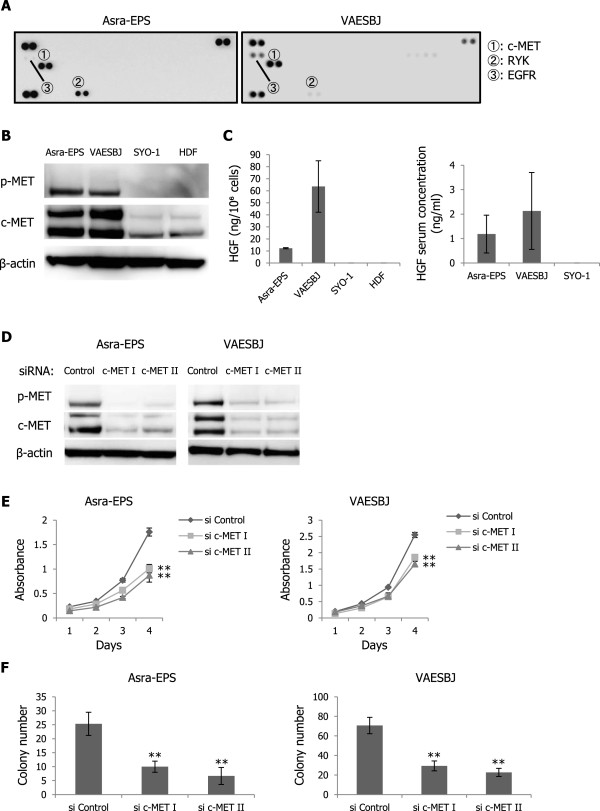
**c-MET is highly activated through an autocrine mechanism in EpS, and c-MET activation affects EpS cell growth. A)** Expression of phosphorylated RTKs in Asra-EPS and VAESBJ cells. **B)** Protein expression of c-MET and p-MET in Asra-EPS, VAESBJ, SYO-1, and HDF cells. **C)** HGF levels in cell-conditioned media and HGF serum concentrations in mice bearing xenograft tumors. Columns, mean; bars, SD. **D)** Expression of c-MET and p-MET in Asra-EPS and VAESBJ cells transfected with anti-c-MET siRNAs or a control siRNA. **E)** Proliferation of Asra-EPS and VAESBJ cells transfected with anti-c-MET siRNAs or a control siRNA. Cells transfected with siRNAs were cultured for 96 hours. Cell viability was determined by WST-1 assay every 24 hours. Points, mean; bars, SD. **, p < 0.01, compared with control. **F)** Colony formation of Asra-EPS and VAESBJ cells transfected with anti-c-MET siRNAs or a control siRNA. Columns, mean; bars, SD. **, p < 0.01, compared with control.

Reportedly, mTOR inhibitors promoted AKT activation by relieving feedback inhibition of RTK signaling [19–22]. To identify potential RTKs crucial for induction of AKT phosphorylation in response to RAD001, we conducted phospho-RTK array analyses and sought driver tyrosine kinase in Asra-EPS and VAESBJ cells. c-MET was markedly phosphorylated in both EpS cell lines (Figure 4A). While c-MET was expressed but not phosphorylated in SYO-1, a human synovial sarcoma cell line, or HDF cells, higher expression and phosphorylation of c-MET were observed in Asra-EPS and VAESBJ cells (Figure 4B). To determine whether c-MET phosphorylation was caused by an HGF autocrine loop in EpS, we examined the expression of cell-secreted HGF by ELISA. As expected, Asra-EPS and VAESBJ cells secreted high levels of HGF into culture media, while SYO-1 or HDF cells did not (Figure 4C). In addition, we also detected high amounts of human HGF in the sera of mice bearing EpS xenograft tumors (Figure 4C). The simultaneous expression of c-MET and HGF and the basal level of p-MET in EpS suggested that the HGF/c-MET signaling pathway was constitutively stimulated through an autocrine mechanism.

To assess the role of the HGF/c-MET pathway in EpS cell growth, we used RNA interference technology *in vitro*. Two kinds of anti-c-MET-specific siRNAs were transfected into EpS cells, which resulted in significant inhibition of c-MET and p-MET expression (Figure 4D). The silencing of c-MET expression decreased the proliferation and colony formation of EpS cells (Figure 4E, F). These findings suggested that the HGF/c-MET signaling pathway affected EpS cell growth.

### INC280 inhibits EpS cell growth, but the dependency of VAESBJ cells on c-MET signaling differs from that of Asra-EPS cells

**Figure 5:**
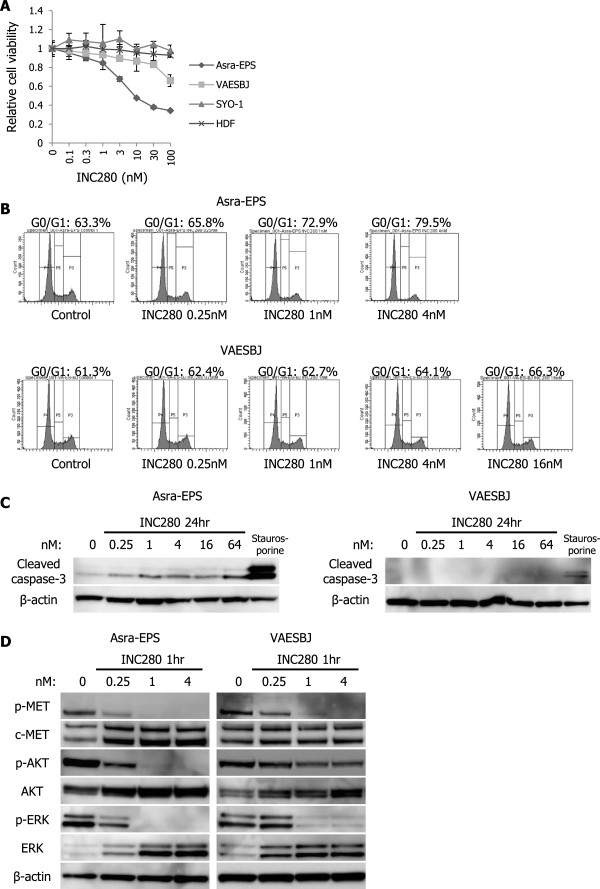
**INC280 inhibits EpS cell growth** ***in vitro*** **, but Asra-EPS and VAESBJ cells differ from each other with regard to dependency on c-MET signaling. A)** Sensitivities of Asra-EPS, VAESBJ, SYO-1, and HDF cells to INC280. Cells were exposed to different concentrations of INC280 for 72 hours. Cell viability was determined by WST-1 assay. Points, mean; bars, SD. **B)** PI staining fluorescence-activated cell sorting analyses of the DNA contents of Asra-EPS and VAESBJ cells in response to INC280. Asra-EPS and VAESBJ cells were incubated with 0.25–4 nM and 0.25–16 nM of INC280 for 24 hours, respectively. **C)** Effects of INC280 on caspase-3 cleavage in Asra-EPS and VAESBJ cells. Cells were treated with 0.25–64 nM of INC280 or vehicle for 24 hours. Staurosporine was used as a positive control. **D)** Effects of INC280 on phosphorylation of c-MET and its downstream effectors in Asra-EPS and VAESBJ cells. Cells were treated with 0.25–4 nM of INC280 or vehicle for 1 hour.

A recently developed c-MET inhibitor, INC280, has been shown to inhibit c-MET-dependent cell motility, proliferation, and invasion in several cancer cell lines *in vitro* and *in vivo* [31]. First, we tested the antitumor effects of INC280 on the growth of Asra-EPS and VAESBJ cells *in vitro*. Proliferation of Asra-EPS and VAESBJ cells was suppressed in a dose-dependent manner by INC280 treatment, albeit with continuous proliferation of SYO-1 or HDF cells (Figure 5A). However, its anti-proliferative effects were much higher on Asra-EPS cells than on VAESBJ cells (Figure 5A). Flow cytometry analyses showed that INC280 induced a greater increase in G0/G1 phase population in Asra-EPS cells than in VAESBJ cells (Figure 5B). Cleavage of caspase-3 was induced after INC280 exposure in a dose-dependent manner in Asra-EPS cells, but its induction was hardly observed in VAESBJ cells (Figure 5C). These data indicated that Asra-EPS cells were more sensitive to INC280 than VAESBJ cells because its effects inducing G0/G1 cell cycle arrest and apoptosis were higher on Asra-EPS cells than on VAESBJ cells.

Second, we examined the efficacy of INC280 on the c-MET downstream pathways in Asra-EPS and VAESBJ cells. The PI3K/AKT/mTOR and mitogen-activated protein kinase (MAPK)/ERK signaling pathways are downstream of c-MET signaling [25]. INC280 strikingly inhibited phosphorylation of c-MET and its downstream molecules such as AKT and ERK in Asra-EPS cells but did not decrease phosphorylation of AKT or ERK in HDF cells (Figure 5D, Additional file 2: Figure S2). These results suggested that Asra-EPS cells were highly addicted to c-MET signaling. By contrast, INC280 remarkably blocked phosphorylation of c-MET and ERK in VAESBJ cells, but AKT phosphorylation was incompletely suppressed (Figure 5D). These data indicated that compared with Asra-EPS cells, VAESBJ cells were less dependent on c-MET signaling *in vitro*, which resulted in sustained AKT activation after INC280 treatment in VAESBJ cells.

**Figure 6:**
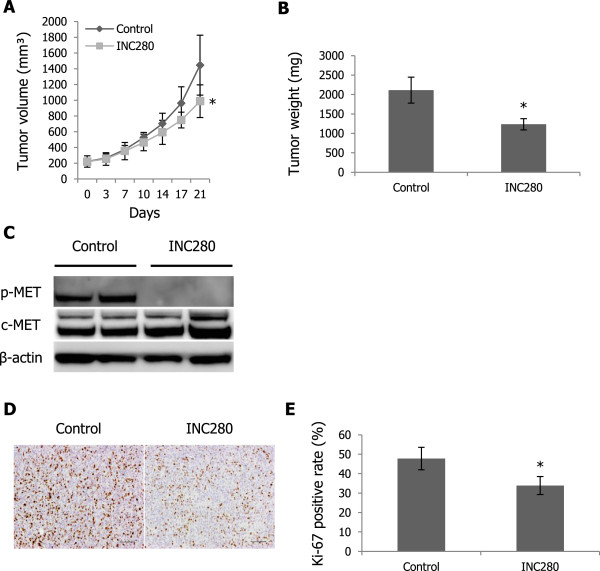
**INC280 delays VAESBJ xenograft tumor growth** ***in vivo*** **. A)** Effects of INC280 on VAESBJ xenograft tumor growth. Mice bearing VAESBJ xenograft tumors were treated with 10 mg/kg INC280 (n = 5) or vehicle control (n = 5) once a day. Points, mean; bars, SD. *, p < 0.05, compared with control treatment. **B)** VAESBJ xenograft tumor weight in control-treated and INC280-treated mice. The average tumor weight recorded at termination of the study was 2112 ± 335 mg in the control group and 1236 ± 145 mg in the INC280 group. Columns, mean; bars, SD. *, p < 0.05, compared with control treatment. **C)** Effects of INC280 on c-MET phosphorylation in VAESBJ xenograft tumors. Tumors were harvested 3 hours after the last administration and then cell lysates were prepared. **D)** Immunohistochemical staining of Ki-67 in control-treated and INC280-treated VAESBJ xenograft tumors. Scale bars: 100 μm. **E)** Ki-67-positivity rate of control-treated and INC280-treated VAESBJ xenograft tumors. Columns, mean; bars, SD. *, p < 0.05, compared with control treatment.

We then evaluated the antitumor effects of INC280 on progression of VAESBJ xenograft tumors in mice. An INC280 dose of 10 mg/kg given orally once a day was selected on the basis of the previous observation that this therapeutic dose resulted in inhibition of c-MET phosphorylation *in vivo* [31]. The administration of INC280 delayed VAESBJ xenograft tumor growth compared with that of the vehicle control, whereas INC280-treated tumors grew gradually (Figure 6A, B). Western blot analyses demonstrated a decrease in expression of phosphorylated c-MET in INC280-treated tumors (Figure 6C). Immunohistochemical studies showed a decrease in the rate of Ki-67-positive tumor cells in INC280-treated tumors compared with that in the control-treated tumors (Figure 6D, E). These results suggested that INC280 treatment exerted limited antitumor effects on VAESBJ xenograft tumor growth by blocking only c-MET signaling.

### Activation of AKT and ERK is enhanced by RAD001 through a c-MET-dependent mechanism in EpS

**Figure 7:**
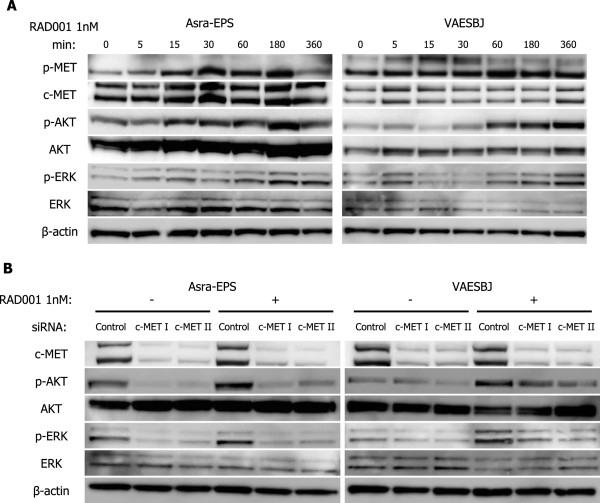
**RAD001-induced activation of AKT and ERK is dependent on c-MET signaling pathway in EpS. A)** Effects of RAD001 on phosphorylation of c-MET and its downstream effectors in Asra-EPS and VAESBJ cells. Cells were treated with 1 nM RAD001 for 5–360 minutes. **B)** Effects of RAD001 on phosphorylation of AKT and ERK in Asra-EPS and VAESBJ cells transfected with anti-c-MET siRNAs or a control siRNA. Cells transfected with siRNAs were treated with 1 nM RAD001 for 1 hour.

Recently, mTOR inhibitors have been shown to increase RTK activity and promote activation of not only AKT but also ERK, which caused intrinsic resistance to mTOR inhibitors [32]. Then, we evaluated the effects of RAD001 on c-MET and its downstream AKT and ERK in EpS. Phosphorylation of c-MET, AKT, and ERK was increased after RAD001 treatment in Asra-EPS and VAESBJ cells (Figure 7A). Furthermore, RAD001-induced phosphorylation of AKT and ERK was attenuated by silencing of c-MET in both EpS cell lines (Figure 7B). These data implied that activation of AKT and ERK was enhanced by mTOR inhibition with RAD001 through a c-MET-dependent mechanism in EpS.

### Combining RAD001 with INC280 remarkably inhibits EpS cell growth *in vitro*

**Figure 8:**
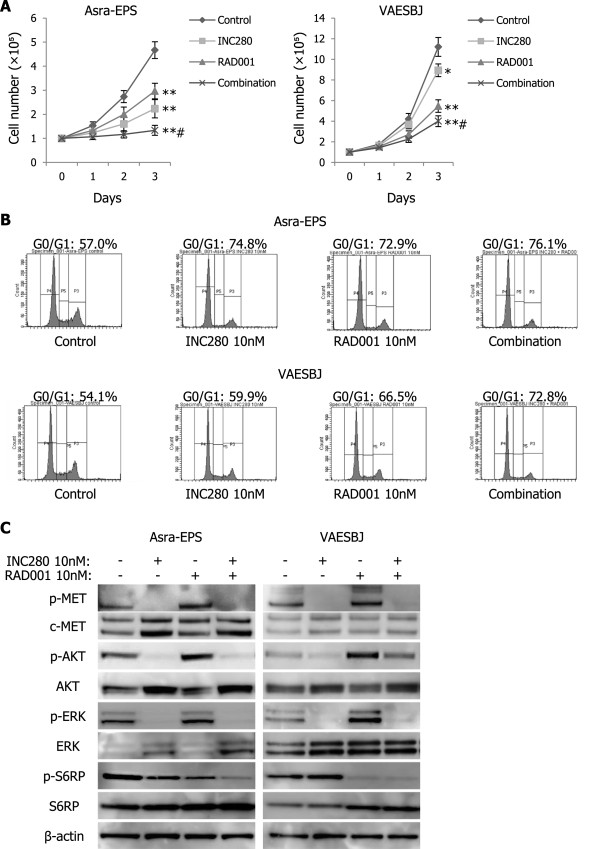
**Combination of RAD001 and INC280 surprisingly inhibits EpS cell growth by blocking the mTOR and c-MET pathways** ***in vitro*** **. A)** Combined efficacy of RAD001 and INC280 on EpS cell proliferation. Cells were treated with 10 nM INC280, 10 nM RAD001, their combination, or vehicle for 72 hours. The cell number was counted every 24 hours. Points, mean; bars, SD. *, p < 0.05, **, p < 0.01, compared with control; #, p < 0.05, compared with INC280 or RAD001 monotherapy. **B)** PI staining fluorescence-activated cell sorting analyses of the DNA contents of Asra-EPS and VAESBJ cells in response to the combination of RAD001 and INC280. Cells were treated with 10 nM INC280, 10 nM RAD001, their combination, or vehicle for 24 hours. **C)** Effects of combined treatment with RAD001 and INC280 on phosphorylation of c-MET and its downstream effectors. Cells were treated with 10 nM INC280, 10 nM RAD001, their combination, or vehicle for 1 hour.

Because RAD001-induced reactivation of AKT and ERK may limit the antitumor effects of RAD001, we investigated the combined efficacy of RAD001 and INC280 on EpS cell growth *in vitro*. Simultaneous administration of both compounds remarkably inhibited the proliferation of Asra-EPS and VAESBJ cells compared with either single agent alone or a control (Figure 8A). Cell cycle analyses revealed the superior effects of inducing G0/G1 cell-cycle arrest by combined treatment with RAD001 and INC280 in both EpS cell lines (Figure 8B). The combination of RAD001 and INC280 blocked RAD001-induced phosphorylation of c-MET, AKT, and ERK in EpS (Figure 8C). In addition, compared with INC280 alone, the combination notably inhibited S6RP phosphorylation (Figure 8C). These data indicated that the combination of RAD001 and INC280 exerted superior antitumor effects on EpS cell growth by blocking both mTOR and c-MET signaling pathways *in vitro*.

### Combined therapy with RAD001 and INC280 significantly abrogates EpS tumor growth *in vivo*

**Figure 9:**
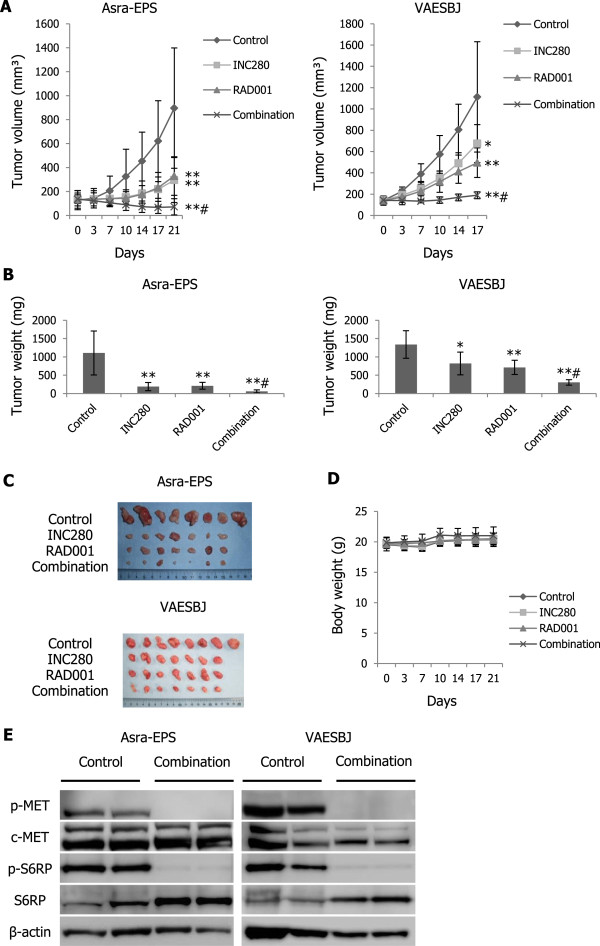
**Combined targeting of mTOR and c-MET results in superior antitumor effects on EpS tumor growth** ***in vivo*** **. A)** Effects of combination therapy with RAD001 and INC280 on EpS xenograft tumor growth. Mice bearing Asra-EPS and VAESBJ xenograft tumors were treated with INC280 (n = 7), RAD001 (n = 7), their combination (n = 7), or vehicle control (n = 8). In the INC280 and combination groups, mice were treated with 10 mg/kg INC280 once a day. In the RAD001 and combination groups, mice were treated with 5 mg/kg RAD001 thrice a week. Points, mean; bars, SD. *, p < 0.05, **, p < 0.01, compared with control treatment; #, p < 0.05, compared with INC280 or RAD001 monotherapy. **B)** Asra-EPS and VAESBJ xenograft tumor weight in the four groups. The average Asra-EPS tumor weights recorded at termination of the study were: control group, 1106 ± 600 mg; INC280 group, 188 ± 113 mg; RAD001 group, 210 ± 93 mg; and combination group, 57 ± 40 mg. The average VAESBJ tumor weights were: control group, 1337 ± 376 mg; INC280 group, 818 ± 311 mg; RAD001 group, 718 ± 195 mg; and combination group, 302 ± 77 mg. Columns, mean; bars, SD. *, p < 0.05, **, p < 0.01, compared with control treatment; #, p < 0.05, compared with INC280 or RAD001 monotherapy. **C)** Appearance of the resected Asra-EPS and VAESBJ xenograft tumors in the four groups. **D)** Body weight of mice bearing Asra-EPS xenograft tumors in the four groups. Points, mean; bars, SD. **E)** Effects of combined therapy with RAD001 and INC280 on phosphorylation of S6RP and c-MET in Asra-EPS and VAESBJ xenograft tumors. Xenograft tumors were harvested 3 hours after the last administration and then cell lysates were prepared.

**Figure 10:**
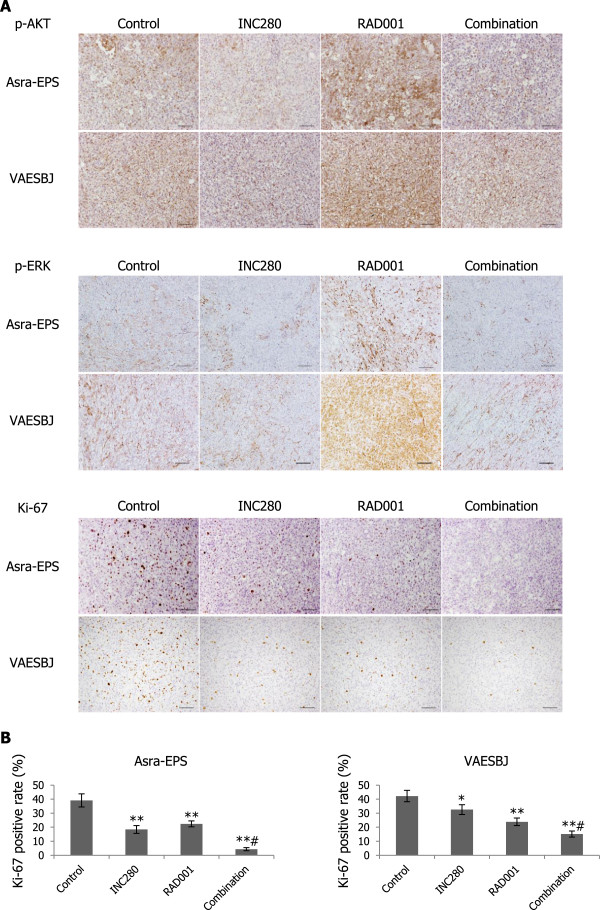
**Combination therapy decreases RAD001-induced activation of AKT and ERK in EpS xenograft tumors. A)** Immunohistochemical staining of p-AKT, p-ERK, and Ki-67 in Asra-EPS and VAESBJ xenograft tumors in the four groups. Scale bars: 100 μm. **B)** Ki-67-positivity rate of Asra-EPS and VAESBJ xenograft tumors in the four groups. Columns, mean; bars, SD. *, p < 0.05, **, p < 0.01, compared with control treatment; #, p < 0.05, compared with INC280 or RAD001 monotherapy.

To assess the antitumor effects of combination therapy with RAD001 and INC280 on EpS xenograft tumor growth, a 4-armed therapeutic study was conducted. Either RAD001 or INC280 as a single agent inhibited Asra-EPS and VAESBJ xenograft tumor growth compared with the vehicle control (Figure 9A–C). Most importantly, combined treatment significantly abrogated EpS xenograft tumor growth compared with the treatment with each single drug alone (Figure 9A–C). No significant body weight loss was observed in drug-treated mice bearing Asra-EPS xenograft tumors (Figure 9D). Western blot analyses showed that phosphorylation of both c-MET and S6RP was blocked in combination-treated tumors (Figure 9E). Immunohistochemical studies showed that phosphorylation of AKT and ERK in Asra-EPS and VAESBJ xenograft tumors was increased by RAD001 treatment but attenuated by the combination therapy with RAD001 and INC280 (Figure 10A, Additional file 3: Figure S3). Moreover, the decrease in the rate of Ki-67-positive staining cells was most pronounced in the combination-treated tumors (Figure 10A, B). These data suggested that combined inhibition of mTOR and c-MET signaling pathways also exhibited significant antitumor effects on EpS *in vivo*.

### AKT and HGF/c-MET signaling pathways are frequently activated in tumors of patients with EpS

To investigate the clinical relevance of AKT and HGF/c-MET pathways in EpS, we examined expression of p-AKT, HGF, c-MET, and p-MET in 6 EpS clinical samples by immunohistochemical analyses. In spite of different expression levels among the clinical samples of EpS patients, p-AKT, HGF, and c-MET were expressed in all 6 EpS samples, and p-MET expression was detected in 5 (83.3%) of 6 samples (Additional file 4: Figure S4, Additional file 5: Table S1). These results indicated that the activation of AKT and HGF/c-MET pathways was frequently observed in tumors of patients with EpS, as in EpS cell lines.

## Discussion

Activation of the AKT/mTOR signaling pathway through mutation of pathway components as well as through activation of upstream signaling molecules occurs in a majority of cancers and contributes to deregulation of proliferation and resistance to apoptosis [6, 7]. Constitutive AKT activation was observed in MRTs characterized by loss of INI-1 expression, and a mechanism for AKT activation may be caused by aberrant activation of the insulin-like growth factor 1 receptor (IGF-1R) pathway [33]. Moreover, AKT signaling was activated via autocrine signaling by insulin and the insulin receptor in INI-1-deficient AT/RTs [34]. In addition, INI-1 loss is observed in the majority of EpS [11, 12] and is responsible for the tumorigenic properties of EpS [35], but it is unknown whether the AKT/mTOR pathway is activated in EpS. In the present study, we demonstrated loss of INI-1 expression and constitutive activation of the AKT/mTOR pathway in two human EpS cell lines, Asra-EPS and VAESBJ. Although the histological phenotypes of MRT, AT/RT, and EpS are different from each other, INI-1 expression is lost, and AKT signaling is activated among these distinct tumors. However, little is known about the relationship between INI-1 deficiency and AKT activation in EpS. Asra-EPS and VAESBJ cell lines can be useful tools to investigate this relationship in EpS.

mTOR inhibitors exert antitumor effects on several cancers in which the AKT/mTOR pathway is hyperactivated, but their effects are frequently modest in clinical trials [17, 18]. Biopsy samples from patients treated with mTOR inhibitors confirmed that AKT reactivation occurred clinically and portended a poorer prognosis [19, 36]. AKT reactivation induced by mTOR inhibition in tumor cells is likely to reduce its antitumor effects by activating pathways that attenuate its effects on proliferation and apoptosis; thus, it is an unexpected and potentially undesirable consequence of mTOR inhibition [19]. Recently, it has been shown that mTOR inhibitors increased upstream RTK activity, which resulted in reactivation of not only AKT but also ERK [32]. Here, we demonstrated that an mTOR inhibitor, RAD001, also inhibited EpS cell proliferation but induced reactivation of both AKT and ERK. These results suggested that blockade of this reactivation could enhance the antitumor effects of mTOR inhibitors on EpS.

It has been reported that mTOR inhibitors induced feedback reactivation of AKT signaling through an IGF-1R-dependent, a platelet-derived growth factor receptor A (PDGFRA)-dependent, or a PDGFRB-dependent mechanism [19–22]. However, phospho-RTK array analyses did not show activation of IGF-1R, PDGFRA, or PDGFRB in EpS. Instead, we found that c-MET was the most highly activated RTK in both EpS cell lines and that reactivation of AKT and ERK by mTOR inhibition was c-MET-dependent in EpS. To the best of our knowledge, this is the first study to show that an mTOR inhibitor induces reactivation of AKT and ERK through a c-MET-dependent mechanism. These results provide a rationale for combining mTOR inhibitors with c-MET inhibitors to treat patients with EpS.

HGF stimulation induces c-MET phosphorylation, which in turn activates multiple downstream pathways, including PI3K/AKT and MAPK/ERK signaling [25, 37]. Combined overexpression of HGF and c-MET have been observed in numerous sarcomas [38–40], and HGF can activate c-MET in an autocrine manner in these tumors. We observed that both HGF and c-MET were also overexpressed in Asra-EPS and VAESBJ cells, indicating that c-MET was aberrantly activated by autocrine HGF stimulation in EpS. Cancer-associated c-MET activation triggers cell growth, survival, invasion, migration, and angiogenesis [41–43]. c-MET inhibitors have shown antitumor efficacy in preclinical studies and are currently being evaluated in human cancer clinical trials [44, 45]. In the present study, a selective c-MET inhibitor INC280 showed antitumor effects on EpS cell growth by blocking activation of AKT and ERK. These data indicated that one mechanism for activation of both AKT and ERK pathways was based on the HGF/c-MET autocrine signaling in EpS. However, the sensitivity of VAESBJ cells to INC280 was modest compared with that of Asra-EPS cells. AKT activation was completely blocked by treatment with INC280 in Asra-EPS cells but not in VAESBJ cells. These results suggested that the dependency of VAESBJ cells on HGF/c-MET signaling may differ from that of Asra-EPS cells.

PTEN counteracts the effects of PI3K on AKT, and loss of PTEN expression mediates AKT activation [30]. PTEN status affected anti-c-MET therapies to glioblastomas in which PTEN protein expression was frequently low or absent, and combining anti-HGF/c-MET therapies with mTOR inhibitors additively inhibited growth of glioblastoma xenografts [46]. Xie and colleagues demonstrated no or reduced PTEN expression in many EpS samples, indicating that PTEN deregulation was a common molecular aberration in human EpS [47]. We found that PTEN expression was much lower in VAESBJ cells than in Asra-EPS or control HDF cells, suggesting that epithelioid sarcomas were heterogeneous malignancies in terms of PTEN expression. Our results indicated that reduction of PTEN expression in VAESBJ cells may contribute to sustained AKT activation after INC280 treatment and result in decreased sensitivity to c-MET inhibitors.

In fact, the growth of EpS was also significantly abrogated by treatment with the combination of RAD001 and INC280 *in vitro* and *in vivo*. Their combination notably inhibited mTOR and c-MET signaling pathways that were hyperactivated in human EpS; thus, we propose a combined therapeutic approach using mTOR and c-MET inhibitors for EpS lacking effective systemic treatment.

In the present study, the expression of proteins related to AKT/mTOR and HGF/c-MET pathways were detected in nearly all EpS clinical samples, as in EpS cell lines. However, expression levels of these proteins were different among the clinical samples of EpS patients. These results in clinical and experimental studies suggested that EpS cells exhibited heterogeneity in dependency on AKT and c-MET pathways within the tumor. The activation of these pathways contributes to the cell proliferation, survival, and resistance to chemotherapies in many cancers [6, 7, 37, 41–43]. Therefore, dual targeting of AKT/mTOR and HGF/c-MET pathways may exert significant antitumor effects on EpS cells in which these pathways are activated and help us to overcome this devastating disease.

## Conclusions

Loss of INI-1 expression and constitutive AKT activation were observed in Asra-EPS and VAESBJ cells. EpS cell proliferation was inhibited by treatment with RAD001 *in vitro* and *in vivo*, but reactivation of AKT and ERK through a c-MET-dependent mechanism occurred after RAD001 treatment in EpS. RAD001 combined with INC280 exhibited significant antitumor effects on EpS cell lines both *in vitro* and *in vivo*. Hence our preclinical data suggest that combined targeting of mTOR and c-MET signaling pathways should be a novel and effective strategy for treatment of human EpS.

## Materials and methods

### Cell lines, reagents, and antibodies

We used two human EpS cell lines, Asra-EPS and VAESBJ. Asra-EPS was established from a primary tumor of the patient with angiomatoid type of EpS in our laboratory as previously described [13]. VAESBJ, which was established from a bone marrow aspirate of the patient with EpS whose tumors metastasized to a bone marrow [14], was purchased from the American Type Culture Collection. HDF cells were purchased from Kurabo. A human synovial sarcoma cell line, SYO-1, was kindly provided by Dr. Ozaki (Okayama University, Okayama, Japan). Cells were grown in Dulbecco’s Modified Eagle Medium (Life Technologies, Carlsbad, CA, USA) supplemented with 10% FBS (Sigma-Aldrich, St. Louis, MO, USA). Cells were cultured in a humidified atmosphere at 37°C in 5% CO_2_.

An mTOR inhibitor, RAD001, and an ATP-competitive selective c-MET inhibitor, INC280, were provided by Novartis Pharma AG. According to the manufacturer’s instructions, RAD001 and INC280 were prepared in dimethyl sulfoxide (DMSO) before being added to cell cultures for *in vitro* studies. The oral RAD001 formulation provided by Novartis Pharma AG (everolimus microemulsion preconcentrate and corresponding placebo) for animal experiments was diluted with water to the optimal concentration just before administration via gavage. INC280 was diluted in 0.5% methylcellulose and 0.1% Tween 80 for *in vivo* experiments.

Antibodies against c-MET (#8198; WB, 1:1000; IHC, 1:300), p-MET (Tyr1234/1235; #3077; WB, 1:1000; IHC, 1:150), AKT (#4691; 1:1000), p-AKT (Ser473; #4060; WB, 1:1000; IHC, 1:50), ERK (#4695; 1:1000), p-ERK (Thr202/Tyr204; #4370; WB, 1:2000; IHC, 1:400), mTOR (#2983; 1:1000), p-mTOR (Ser2448; #5536; 1:1000), S6RP (#2217; 1:1000), p-S6RP (Ser235/236; #2211; 1:1000), PTEN (#9188; 1:1000), cleaved caspase-3 (#9661; 1:1000), and beta-actin (#4970; 1:1000) were purchased from Cell Signaling Technology, Inc. (Danvers, MA, USA). An antibody against Ki-67 (M7240; 1:50) was purchased from Dako (Glostrup, Denmark). An antibody against INI-1 (612110; 1:500) was purchased from Becton Dickinson Biosciences (BD Biosciences; San Jose, CA, USA). An antibody against HGF (AF-294-NA; 10 μg/ml) was purchased from R&D systems (Minneapolis, MN, USA). Horseradish peroxidase (HRP)-conjugated secondary antibodies were purchased from GE Healthcare Life Sciences (Piscataway, NJ, USA).

### Patients

Six patients with EpS (5 males and 1 female) were operated in Osaka University Hospital from 1998 to 2012. The mean age at the operation was 59.5 years (49 to 67). Tumor specimens were obtained with the patients’ informed consent and used for immunohistochemical studies.

### Western blot analysis

For the lysate preparation, cells were first washed with PBS and lysed in RIPA buffer (Thermo Scientific, Waltham, MA, USA). Protein concentrations were determined according to the bicinchoninic acid method (Thermo Scientific). Then, the cell lysates were separated on 4%–12% Bis-Tris gels (Life Technologies) and transferred to polyvinylidene difluoride (PVDF) membranes (Nippon Genetics, Tokyo, Japan). The membranes were incubated in 5% skim milk in TBS with Tween 20 (TBS-T) at room temperature. Blocked membranes were incubated with primary antibodies at 4°C overnight, followed by incubation with secondary antibodies at room temperature for 1 hour. After washing in TBS-T, immunoreactive bands were visualized by enhanced chemiluminescence (ECL; GE Healthcare Life Sciences).

### WST-1 cell proliferation assay

Cells were seeded at a density of 1 × 10^3^ cells/well in 96-well plates for cell proliferation assays. The cells were incubated overnight and treated with various concentrations of drugs or vehicle (DMSO) for drug experiments. Cell viability was assessed using the Premix WST-1 cell proliferation assay system (Takara Bio, Inc., Otsu, Japan). Using a microplate reader, absorbance measurements read at 690 nm were subtracted from those read at 450 nm. Relative cell viability was expressed as (absorbance of treated cells minus absorbance of cell-free control)/(absorbance of untreated control minus absorbance of cell-free control).

### Cell cycle analysis

EpS cells were seeded at a density of 5 × 10^5^ cells/dish in 10-cm culture dishes and grown overnight, followed by treatment with RAD001, INC280, their combination, or vehicle. After 24-hour treatment, the cells were collected and stained with propidium iodide (PI) solution (25 μg/ml PI, 0.03% NP-40, 0.02 mg/ml RNase A, 0.1% sodium citrate) for 30 minutes at room temperature. The cell cycle was analyzed using BD FACSCanto II flow cytometer (BD Biosciences).

### *In vivo*animal xenograft models

Five-week-old athymic nude mice (BALB/c nu/nu; SLC, Shizuoka, Japan) were housed at the Institute of Experimental Animal Sciences Osaka University Medical School, in accordance with a guideline approved by the Institutional Animal Care and Use Committee of the Osaka University Graduate School of Medicine. For the xenograft tumor growth assay, 1 × 10^7^ EpS cells were injected subcutaneously into the left side of the back. Therapy was initiated after tumor establishment (>5 mm in the longest diameter). RAD001 and INC280 were administered orally thrice a week and once a day, respectively. Xenograft tumor volume and mice body weight were measured twice a week. Tumor volume was measured with a caliper and calculated according to the formula (A × B^2^)/2, with A being the longest diameter and B the shortest diameter of the tumor. Mice were sacrificed when the total tumor burden reached 2 cm^3^, and the tumor weight was then measured. The tumors were resected for western blot analyses and immunohistochemical studies.

### Immunohistochemistry

Specimens of tumors formed in nude mice and those of patients’ primary tumors were fixed in 10% neutral-buffered formalin, embedded in paraffin, and sectioned in 4-μm thicknesses. Paraffin-embedded sections were deparaffinized and dehydrated. Antigens were retrieved at 95°C for 10 minutes in a 10-mM citrate buffer. After blocking of endogenous peroxidase activity for 10 minutes with methanol containing 3% H_2_O_2_, the sections were reacted for 1 hour with TBS containing 2% bovine serum albumin at room temperature. The sections were incubated with primary antibodies at 4°C overnight. On the next day, sections were incubated for 1 hour with secondary antibodies and stained with 3,3’-diaminobenzidine tetrahydrochloride (DAB; Dako). The sections were finally counterstained with hematoxylin. Immunohistochemical protein expression levels were determined using NIS-Elements software (Nikon Corporation, Tokyo, Japan). Staining of p-AKT, HGF, c-MET, and p-MET in patients’ clinical samples was scored as follows: 0, undetectable (0% positive cells); 1+, focally positive (<10% positive cells); 2+, moderately positive (<50% positive cells), and 3+, intensely positive (more than 50% positive cells). Immunohistochemical results were interpreted as negative (0, 1+) or positive (2+, 3+).

### Phospho-RTK array

To evaluate expression of phosphorylated RTKs, the phospho-RTK array was performed with the Proteome Profiler Array Kit (R&D Systems), according to the manufacturer’s protocol. In brief, the array membrane was blocked for 1 hour, incubated with cell lysates overnight, and then treated with HRP-conjugated anti-phospho-tyrosine antibody for 2 hours at room temperature. The membrane was developed with ECL detection reagent, and RTK spots were visualized.

### ELISA

Cells were cultured at a density of 1 × 10^5^ cells/well in 6-well plates. On the 4^th^ day, cell culture supernatants were collected. When xenograft tumors reached 2 cm^3^, whole blood samples were collected by intracardiac puncture, and sera were obtained. HGF concentrations in cell-conditioned media or sera of xenografted mice were determined by ELISA using a Human HGF Quantikine ELISA kit (R&D Systems), according to the manufacturer’s instruction.

### siRNA transfection

EpS cells were seeded at a density of 3 × 10^5^ cells/well in 6-well plates and grown overnight. Cells were transfected with 20 nM siRNAs for 48 hours using Lipofectamine 2000 (Life Technologies). Two kinds of siRNAs targeting c-MET (constructs I and II; #6618 and #6622) and mTOR (construct I and II; #6381 and #6556), and a non-targeting siRNA (#6568) were purchased from Cell Signaling Technology, Inc.

### Soft agar colony formation assay

Five thousand EpS cells were suspended in 1 ml of 0.5% SeaPlaque Agarose (Lonza, Basel, Switzerland) with normal growth medium and seeded over a basal layer of 0.6% agarose in 35-mm culture dishes. The number of colonies (>100 μm in diameter) per well was counted under a light microscope two weeks later.

### Determination of cell number

EpS cells were plated at a density of 1 × 10^5^ cells/well into 6-well plates and grown overnight before treatment with RAD001, INC280, their combination, or vehicle for 72 hours. Cells were trypsinized with 0.25% trypsin plus EDTA (Life Technologies), and a hemocytometer was used to count the cell number for each well every 24 hours.

### Statistical analysis

Each experiment was performed in triplicate. All data are expressed as means ± SDs. Student’s *t*-test for biological assays and Mann–Whitney’s *U* test for animal experiments were used to evaluate the significance of differences. Values of p < 0.05 were considered statistically significant.

## Electronic supplementary material

Additional file 1: Figure S1: A) Expression of mTOR and p-mTOR in VAESBJ and Asra-EPS cells transfected with anti-mTOR siRNAs or a control siRNA. B) Relative cell viability of VAESBJ cells transfected with anti-mTOR siRNAs or a control siRNA. Cells transfected with siRNAs were cultured for 72 hours. Cell viability was determined by WST-1 assay. Relative cell viability was normalized against cells transfected with a non-targeting siRNA. Columns, mean; bars, SD. *, p < 0.05, **, p < 0.01, compared with control. C) Sensitivities of VAESBJ cells transfected with anti-mTOR siRNAs or a control siRNA to various concentrations of RAD001. Cell viability was measured 72-hour after RAD001 treatment using the WST-1 assay. Relative cell viability was normalized against drug-untreated cells transfected with a non-targeting siRNA. Points, mean; bars, SD. (PDF 113 KB)

Additional file 2: Figure S2: Effects of INC280 on phosphorylation of AKT and ERK in HDF cells. The cells were treated with 10 nM INC280 or vehicle for 1 hour. (PDF 96 KB)

Additional file 3: Figure S3: A) Relative expression levels of p-AKT in Asra-EPS and VAESBJ xenograft tumors in the four groups using NIS-Elements software (Nikon Corporation). Relative expression levels were normalized against control-treated tumors. Columns, mean; bars, SD. *, p < 0.05. B) Relative expression levels of p-ERK in Asra-EPS and VAESBJ xenograft tumors in the four groups. Relative expression levels were normalized against control-treated tumors. Columns, mean; bars, SD. *, p < 0.05. (PDF 128 KB)

Additional file 4: Figure S4: Immunohistochemical expression of p-AKT, HGF, c-MET, and p-MET in 6 EpS clinical samples. Scale bars: 100 μm. (PDF 452 KB)

Additional file 5: Table S1: Scoring of p-AKT, HGF, c-MET, and p-MET staining in patients’ clinical samples. Scores of 0 or 1+ were defined as negative and those of 2+ or 3+ as positive. (PDF 91 KB)

## Abbreviations

AT/RT: Atypical teratoid/rhabdoid tumor
EpS: Epithelioid sarcoma
HGF: Hepatocyte growth factor
HDF: Human dermal fibroblast
IGF-1R: Insulin-like growth factor 1 receptor
INI-1: Integrase interactor 1
MRT: Malignant rhabdoid tumor
MAPK: Mitogen-activated protein kinase
PI3K: Phosphatidylinositol 3-kinase
PDGFR: Platelet-derived growth factor receptor
RTK: Receptor tyrosine kinase
S6RP: S6 ribosomal protein.
