# Supplementary figures and images for: Combined targeting of mTOR and c-MET signaling pathways for effective management of epithelioid sarcoma
Source: Mol Cancer. 2014 Aug 7;13:185. doi: 10.1186/1476-4598-13-185 (PMC4249599; doi:10.1186/1476-4598-13-185)

Additional file 1: Figure S1

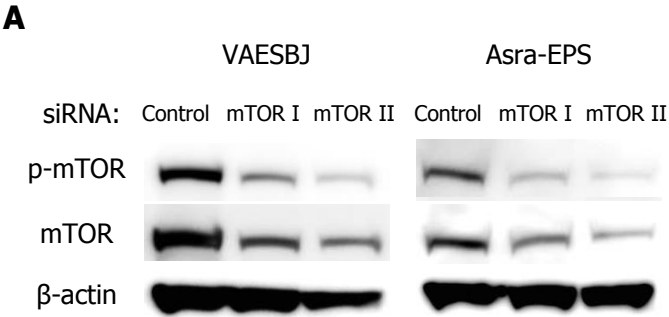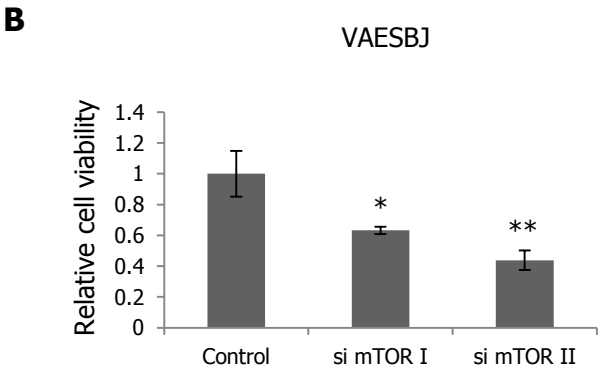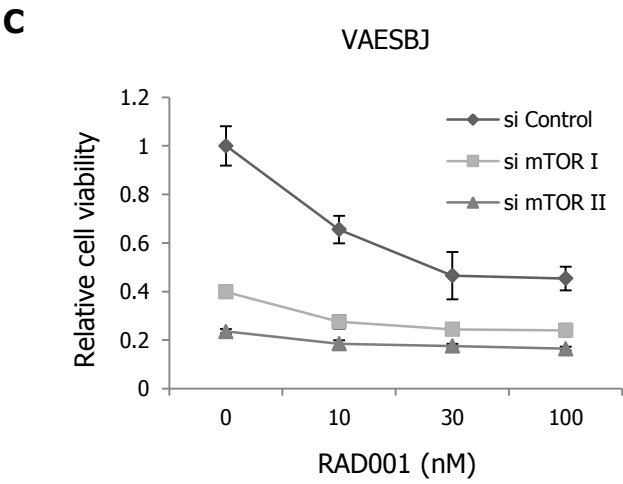

Supplement: Supplementary file 1 — Additional file 1: Figure S1: A) Expression of mTOR and p-mTOR in VAESBJ and Asra-EPS cells transfected with anti-mTOR siRNAs or a control siRNA. B) Relative cell viability of VAESBJ cells transfected with anti-mTOR siRNAs or a control siRNA. Cells transfected with siRNAs were cultured for 72 hours. Cell viability was determined by WST-1 assay. Relative cell viability was normalized against cells transfected with a non-targeting siRNA. Columns, mean; bars, SD. *, p < 0.05, **, p < 0.01, compared with control. C) Sensitivities of VAESBJ cells transfected with anti-mTOR siRNAs or a control siRNA to various concentrations of RAD001. Cell viability was measured 72-hour after RAD001 treatment using the WST-1 assay. Relative cell viability was normalized against drug-untreated cells transfected with a non-targeting siRNA. Points, mean; bars, SD. (PDF 113 KB) [file 12943_2014_1387_MOESM1_ESM.pdf]

**Additional file 2: Figure S2**

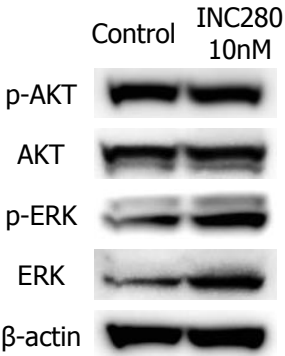

Supplement: Supplementary file 2 — Additional file 2: Figure S2: Effects of INC280 on phosphorylation of AKT and ERK in HDF cells. The cells were treated with 10 nM INC280 or vehicle for 1 hour. (PDF 96 KB) [file 12943_2014_1387_MOESM2_ESM.pdf]

Additional file 3: Figure S3

A

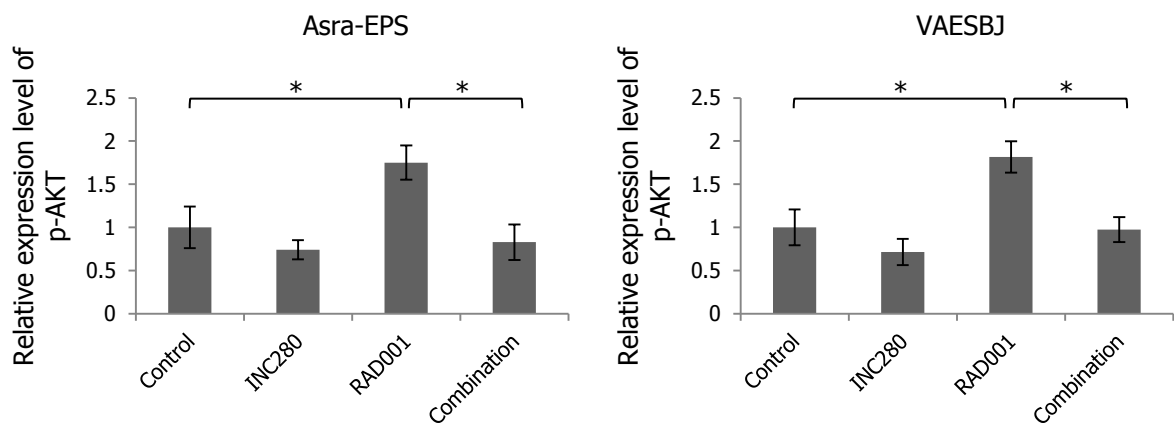

B

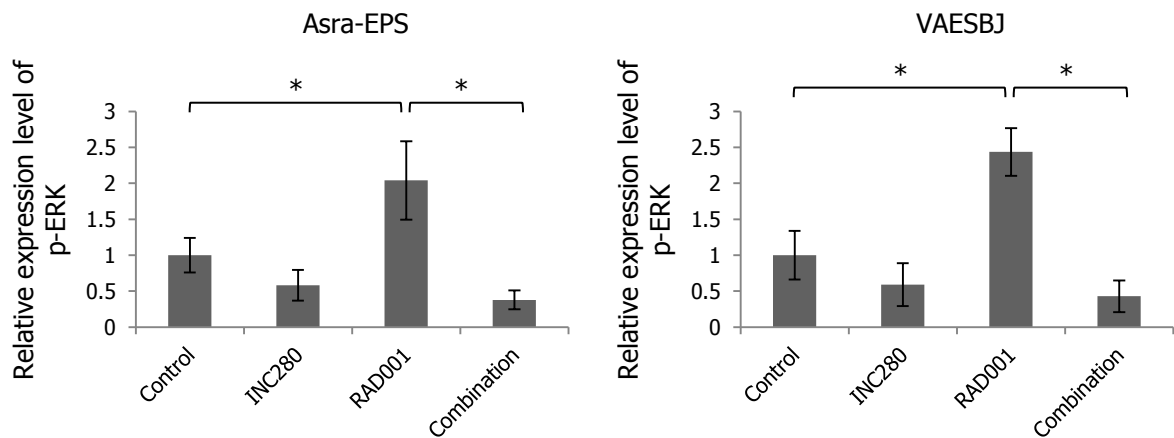

Supplement: Supplementary file 3 — Additional file 3: Figure S3: A) Relative expression levels of p-AKT in Asra-EPS and VAESBJ xenograft tumors in the four groups using NIS-Elements software (Nikon Corporation). Relative expression levels were normalized against control-treated tumors. Columns, mean; bars, SD. *, p < 0.05. B) Relative expression levels of p-ERK in Asra-EPS and VAESBJ xenograft tumors in the four groups. Relative expression levels were normalized against control-treated tumors. Columns, mean; bars, SD. *, p < 0.05. (PDF 128 KB) [file 12943_2014_1387_MOESM3_ESM.pdf]

**Additional file 4: Figure S4**

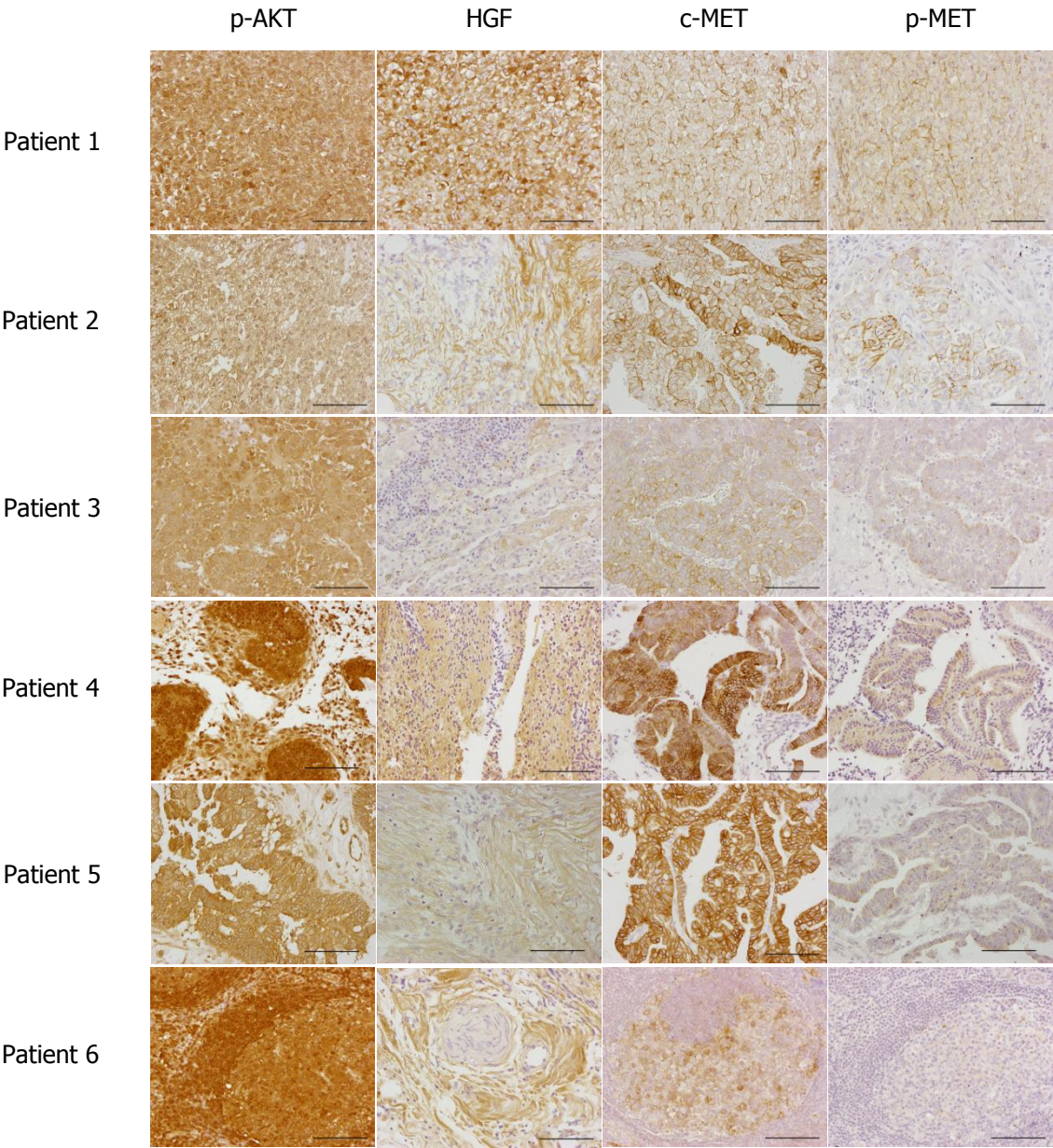

Supplement: Supplementary file 4 — Additional file 4: Figure S4: Immunohistochemical expression of p-AKT, HGF, c-MET, and p-MET in 6 EpS clinical samples. Scale bars: 100 μm. (PDF 452 KB) [file 12943_2014_1387_MOESM4_ESM.pdf]
