# Supplementary material for: Combined targeting of mTOR and c-MET signaling pathways for effective management of epithelioid sarcoma
Source: Mol Cancer. 2014 Aug 7;13:185. doi: 10.1186/1476-4598-13-185 (PMC4249599; doi:10.1186/1476-4598-13-185)
Supplement: Supplementary file 5 — Additional file 5: Table S1: Scoring of p-AKT, HGF, c-MET, and p-MET staining in patients’ clinical samples. Scores of 0 or 1+ were defined as negative and those of 2+ or 3+ as positive. (PDF 91 KB) [file 12943_2014_1387_MOESM5_ESM.pdf]

Additional file 5: Table S1

Patient	p-AKT	HGF	c-MET	p-MET
1	3+	3+	3+	3+
2	3+	3+	3+	2+
3	3+	2+	3+	2+
4	3+	3+	3+	3+
5	3+	3+	3+	2+
6	3+	3+	2+	1+

2+, 3+: positive staining

0, 1+: negative staining
